# Informational Postcards Increase Engagement with Remote Monitoring Among Veterans with Pacemakers and Implantable Cardioverter-Defibrillators: a Stepped-Wedge Randomized Controlled Trial

**DOI:** 10.1007/s11606-023-08478-9

**Published:** 2024-01-22

**Authors:** Megan M. McLaughlin, Merritt H. Raitt, Gary Tarasovsky, Mary A. Whooley, Sanket S. Dhruva

**Affiliations:** 1 San Francisco Veterans Affairs Health Care System, San Francisco, CA USA; 2 Department of Medicine, University of California San Francisco School of Medicine, San Francisco, CA USA; 3 Portland Veterans Affairs Health Care System, Portland, OR USA

**Keywords:** remote monitoring, pacemakers, implantable cardioverter-defibrillators, ICD, adherence

## Abstract

**Background:**

Remote monitoring (RM) of pacemakers and implantable cardioverter-defibrillators (ICDs) reduces morbidity and mortality. However, many patients are not adherent to RM.

**Objective:**

To test the effect of informational postcards on RM adherence.

**Design/Patients:**

Stepped-wedge randomized controlled trial among Veterans with pacemakers and ICDs.

**Intervention:**

In wave 1, Veterans who had sent at least 1 transmission within the past 2 years but had become non-adherent were randomly assigned to receive a postcard or no postcard. Those receiving postcards were randomized to 1 of 2 messages: (1) a”warning” postcard describing risks of non-adherence or (2) an “encouraging” postcard describing benefits of adherence. In wave 2, Veterans who had either not received a postcard in wave 1 or had since become non-adherent were mailed a postcard (again, randomized to 1 of 2 messages). Patients who did not send an RM transmission within 1 month were mailed a second, identical postcard.

**Main Measures:**

Transmission within 70 days.

**Key Results:**

Overall, 6351 Veterans were included. In waves 1 and 2, postcards were mailed to 5657 Veterans (2821 “warning” messages and 2836 “encouraging” messages). Wave 1 included 2178 Veterans as controls (i.e., not mailed a postcard), some of whom received a postcard in wave 2 if they remained non-adherent. In wave 2, 3473 postcards were sent. Of the 5657 patients mailed a postcard, 2756 (48.7%) sent an RM transmission within 70 days, compared to 530 (24.3%) of 2178 controls (absolute difference 24.4%, 95% confidence interval [CI] 22.2%, 26.6%). Of those who sent a transmission, 71.8% did so after the first postcard. Transmission rates at 70 days did not significantly differ between “warning” and “encouraging” messages (odds ratio 1.04, 95% CI 0.92, 1.18).

**Conclusions:**

Informational postcards led to a 24.4% absolute increase in adherence at 70 days among Veterans with pacemakers and ICDs who were non-adherent to RM.

**Supplementary Information:**

The online version contains supplementary material available at 10.1007/s11606-023-08478-9.

## Introduction

Remote monitoring (RM) of pacemakers and implantable cardioverter-defibrillators (ICDs) is the process by which data about device function, arrhythmias, and other physiologic parameters is transmitted to clinicians.^1^ RM is a Class 1, Level of Evidence A recommendation.^1, 2^ RM reduces time to clinical decisions or event detection,^3–5^ inappropriate ICD shocks,^3, 6^ hospitalizations and emergency department visits,^4, 7, 8^ and mortality.^8–11^ RM also decreases the need for in-person visits and has outcomes comparable to office follow-up;^3, 12–15^ these are reasons why RM lowers costs^7, 12, 13, 16, 17^ and improves patient satisfaction.^18^ During the COVID-19 pandemic, RM was recommended to reduce the need for in-person visits.^19–24^

To achieve the clinical benefits of RM, patients with pacemakers or ICDs must adhere to sending routine RM transmissions, which are generally done approximately every 90 days. Unfortunately, adherence rates are low.^10, 25, 26^ In a national Veterans Health Administration (VA) study, over a 2-year period the average RM adherence among Veteran patients was 71.9%, and only 30.9% had complete adherence (complete adherence meaning that 100% of their maximum adherence period was covered by a remote transmission).^27^ These are similar or better than rates in non-VA patient populations,^10, 25^ but still represent an important opportunity for improvement.

Therefore, effective strategies to improve adherence to RM are needed. Historically, clinicians have reminded patients at in-person visits and called patients who have become non-adherent, which is time-consuming^28^ and may decrease as a priority with competing clinical responsibilities.^29^ There have been no published studies of a reminder strategy that involves direct-to-patient contact to improve RM adherence. However, direct-to-patient contact has been demonstrated in other settings to improve uptake of care such as vaccinations and cancer screening.^30–33^

Additionally, the content and framing may affect the effectiveness of the strategy. Message framing involves changing how a message is presented without changing the content of the message or the incentives, and it can affect risk perceptions and health behaviors.^34, 35^ Behavioral economics and psychology research have examined whether there is a difference in outcomes between messages highlighting the positive outcomes of performing a behavior (gain-framing) versus the negative outcomes of not performing a behavior (loss-framing).^34–37^ In the healthcare context, loss-framing and gain-framing have shown mixed results in previous studies. A meta-analysis of gain-framed vs. loss-framed messages showed no significant difference in vaccination rates between the two messaging strategies.^38^ The same authors found that in the context of disease detection, loss-framed messages had a statistically significant but small advantage over gain-framed messages for breast cancer screening but not for other types of screening.^39^ Conversely, for disease prevention behaviors, gain-framed messages were more effective than loss-framed messages for dental hygiene behaviors but not for other disease prevention behaviors.^40^

Accordingly, we sought to evaluate the effect of mailing informational postcards with two different messaging strategies on RM adherence among Veterans who were non-adherent.

## METHODS

### Study Population

The study included all Veteran patients with wireless RM-capable pacemakers and ICDs followed by the VA National Cardiac Device Surveillance Program (VANCDSP). All patients had previously sent at least 1 remote transmission within the past 2 years but had become non-adherent (defined as missing their last scheduled transmission by at least 10 days). Patients who transmit on time but then miss a transmission become non-adherent when they miss a transmission, until they send another transmission. The study was conducted from July 2020 to December 2021. This was a quality improvement project conducted in partnership with the VA Measurement Science Quality Enhancement Research Initiative and the VANCDSP. The National Clinical Trial (NCT) number is NCT06068699.

### Intervention

**Figure 1:**
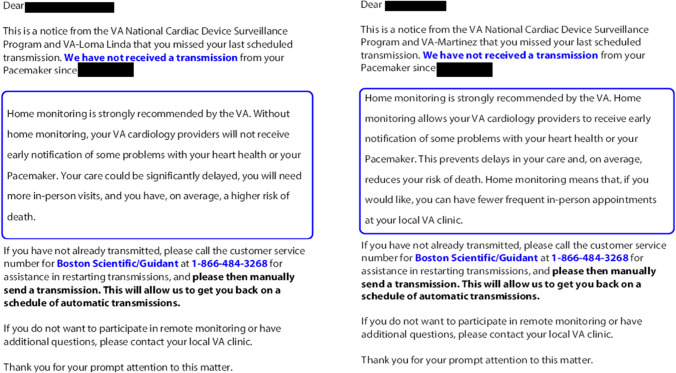
Example “warning” (left) and “encouraging” (right) postcards.

We designed two informational postcards: (1) a “warning” postcard describing risks of non-adherence to RM (loss-frame) and (2) an “encouraging” postcard that framed the same content with a positive/beneficial message (gain-frame) (Fig. 1). For example, the “warning” postcard stated “Without home monitoring, your VA cardiology providers will not receive early notification of some problems with your heart health or your pacemaker. Your care could be significantly delayed, you will need more in-person visits, and you have, on average, a higher risk of death.” Both postcards included the date of the patient’s last RM transmission, a request to send a manual transmission (which the patient can generally do themselves through their remote monitor, although processes differ across devices), and the customer service number for the manufacturer of their pacemaker or ICD. As patients with pacemakers and ICDs manufactured by Biotronik cannot manually send a transmission, after the initial mailing, this request was removed for patients with Biotronik devices after this sentence had been inadvertently included on the initial postcard. Patients with Biotronik pacemakers and ICDs would only have been able to call the manufacturer’s customer service number after the first postcard, and all subsequent postcards instructed patients with Biotronik devices to call the manufacturer for assistance in restarting transmissions.

**Figure 2:**
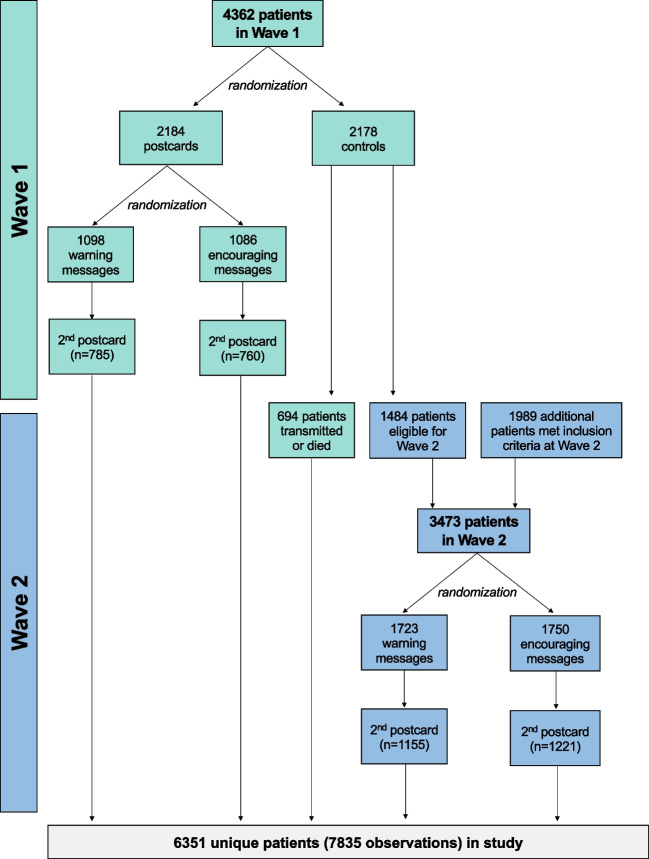
Study flow chart.

To study the effect of these postcards, we conducted a stepped-wedge randomized controlled trial (RCT) with two waves (Fig. 2). We used simple randomization in both waves. The first wave included all patients who were eligible based on non-adherence to RM. Patients in this first wave were randomized 1:1 to receive a postcard by mail or no postcard (control). Patients who received a postcard were further randomized 1:1 to receive either of the two messages (“warning” vs. “encouraging”). Patients who were controls in the first wave were eligible to participate in the second wave if they remained non-adherent and followed by VA. We also reassessed eligibility prior to the start of wave 2 and included in this second wave additional patients who had become newly eligible based on non-adherence since the first wave patients, as has been described in other stepped-wedge trials.^41^ In the second wave, all patients received a postcard, again randomized 1:1 to the two messages. In both waves, patients who did not send an RM transmission within one month were mailed a second, identical postcard.

### Data Sources and Covariates

Data were derived from two sources: (1) the VANCDSP, which includes all patients who are followed by VA for their pacemaker or ICD, and (2) the VA Corporate Data Warehouse (CDW), which contains patient-level electronic health record data, including demographics as well as outpatient and inpatient encounters.

Device-related data obtained from the VANCDSP were device type (pacemaker or ICD), last in-person device interrogation date, implantation date, last remote transmission date, and generator manufacturer. Data obtained from the CDW were age, sex, race, ethnicity, geographic region, rural vs. urban residence, marital status, and both cardiovascular and non-cardiovascular comorbidities. Comorbidities were defined as at least two outpatient or one inpatient diagnosis code (International Classification of Diseases 10th Revision, ICD-10) within the 2 years before or during the study period (Supplementary Table 1).

### Study Outcomes

The primary outcome was RM adherence, defined as sending a single transmission within 70 days of the initial postcard mailing in each wave. We chose 70 days to provide sufficient time for patients to receive replacement RM equipment, if needed. As a secondary outcome, we examined the proportion of patients who transmitted after receiving one postcard vs. two postcards.

### Statistical Analysis

We examined differences in baseline characteristics by randomization arm in wave 1 and in wave 2 using standardized mean differences, with a cutoff of < 0.1 denoting successful randomization.

Because there was a slight delay between identification of patients who qualified for study inclusion and the mailing of postcards (10 days in wave 1 and 11 days in wave 2), we excluded patients who were randomized but transmitted or died prior to the postcard mailing in each wave. We used an intention-to-treat (ITT) approach for the primary analysis. We used logistic regression with generalized estimating equations (GEEs) to compare the proportion of patients who sent a transmission within 70 days of the initial postcard mailing by randomization arm. GEEs were used to account for repeated measures among patients who were controls in wave 1 and subsequently received a postcard in wave 2. We used post-estimation testing to assess whether there was a significant difference between the “warning” vs. “encouraging” messages. All models controlled for study wave number.

We also performed an as-treated analysis, excluding patients whose postcards were returned as undeliverable. In a sensitivity analysis, we excluded patients who changed to non-active status (indicating they died, transferred their device care outside of VA, or newly expressed a desire not to participate in RM) during the study period. In a secondary analysis, we examined the proportion of patients who transmitted after one vs. two postcards.

We conducted subgroup analyses to evaluate for possible differences in the effect of each postcard message compared to controls. We examined subgroups of key demographic factors (age, sex, race, rural vs. urban residence), common cardiovascular comorbidities (heart failure and atrial fibrillation), and comorbidities associated with lower RM adherence in prior research^27^ (dementia, depression, post-traumatic stress disorder [PTSD]). Given that clinicians have the opportunity to check RM adherence during in-person device interrogations, we also examined subgroups by in-person interrogations in the last year. Finally, we hypothesized that patients with a longer time since last transmission may have been less likely to respond to postcards and, thus, examined subgroups of this variable.

We calculated 95% confidence intervals (CIs) for odds ratios and absolute differences. A *p*-value of < 0.05 was considered statistically significant. Statistical analyses were performed using RStudio 2022.07.2.

## Results

### Baseline Characteristics

Of 58,731 patients with pacemakers and ICDs actively followed by the VANCDSP as of July 24, 2020, a total of 6351 unique patients were included. Because some Veterans who were controls (i.e., did not receive a postcard) in wave 1 were subsequently randomized to receive a postcard in wave 2, there were a total of 7835 observations. Of these observations, 5657 were randomized to receive a postcard (2821 with “warning” messages and 2836 with “encouraging” messages), including 1484 patients who were controls in wave 1. There were 2178 controls in wave 1.

Among the total study population of 6351 patients, median age was 70.8 (interquartile range [IQR], 65.2 to 76.6) years. Most patients (97.6%) were male. Overall, 74.5% were White, 17.2% were Black, and 3.6% were of Hispanic or Latinx ethnicity. About half (49.4%) were married, and 38.3% lived in rural areas. A total of 80.0% had coronary artery disease, 75.0% heart failure, and 58.8% atrial fibrillation. Most patients had an ICD (62.8%), and 37.2% had a pacemaker. About two-thirds of patients (65.8%) had an in-person interrogation in the past year. The median time since the last transmission was 209 (IQR, 145 to 379) days.

Baseline characteristics were well-balanced by randomization arm in both waves (Tables 1 and 2), with all standardized mean differences < 0.1.

**Table 1:** Baseline Characteristics of Patient Population in Wave 1, by Randomization Arm

Characteristics	Mailed warning postcard (*n* = 1098)	Mailed encouraging postcard (*n* = 1086)	Controls (no postcard) (*n* = 2178)	Standardized mean difference^a^
Demographic characteristics				
Age, median [IQR], years	70.7 [65.3, 76.9]	70.4 [64.1, 75.9]	70.8 [65.3, 76.8]	0.049
Male sex, *n* (%)	1072 (97.6%)	1049 (96.6%)	2126 (97.6%)	0.037
Race, *n* (%)^b^				0.050
American Indian or Alaska native	10 (0.9%)	8 (0.7%)	16 (0.7%)	
Asian	5 (0.5%)	2 (0.2%)	6 (0.3%)	
Black	183 (16.7%)	209 (19.2%)	373 (17.1%)	
Native Hawaiian or other Pacific Islander	8 (0.7%)	10 (0.9%)	11 (0.5%)	
White	819 (74.6%)	780 (71.8%)	1623 (74.5%)	
Ethnicity, *n* (%)^b^				0.034
Hispanic/Latinx	48 (4.4%)	43 (4.0%)	77 (3.5%)	
Not Hispanic/Latinx	1018 (92.7%)	993 (91.4%)	2025 (93.0%)	
Geographic region, *n* (%)^b,c^				0.029
Northeast	119 (10.8%)	118 (10.9%)	225 (10.3%)	
Midwest	262 (23.9%)	232 (21.4%)	487 (22.4%)	
South	501 (45.6%)	515 (47.4%)	1043 (47.9%)	
West	216 (19.7%)	218 (20.1%)	421 (19.3%)	
Rural residence, *n* (%)^b^	416 (37.9%)	414 (38.1%)	831 (38.2%)	0.002
Marital status, *n* (%)^b^				0.011
Married	543 (49.5%)	504 (46.4%)	1052 (48.3%)	
Divorced/separated	352 (32.1%)	389 (35.8%)	739 (33.9%)	
Never married/single	86 (7.8%)	90 (8.3%)	170 (7.8%)	
Widow/widower	113 (10.3%)	100 (9.2%)	211 (9.7%)	
Co-morbidities, *n* (%)				
Coronary artery disease	902 (82.1%)	860 (79.2%)	1749 (80.3%)	0.009
Heart failure	819 (74.6%)	829 (76.3%)	1660 (76.2%)	0.018
Atrial fibrillation	644 (58.7%)	623 (57.4%)	1287 (59.1%)	0.022
Stroke	211 (19.2%)	196 (18.0%)	431 (19.8%)	0.029
Dementia	151 (13.8%)	146 (13.4%)	305 (14.0%)	0.012
Depression	451 (41.1%)	424 (39.0%)	903 (41.5%)	0.028
Post-traumatic stress disorder	218 (19.9%)	229 (21.1%)	449 (20.6%)	0.004
Alcohol use disorder	143 (13.0%)	139 (12.8%)	282 (12.9%)	0.001
Cancer	241 (21.9%)	214 (19.7%)	463 (21.3%)	0.010
Pacemaker or ICD characteristics				
Device type, *n* (%)				0.027
Pacemaker	399 (36.3%)	368 (33.9%)	793 (36.4%)	
ICD	699 (63.7%)	718 (66.1%)	1385 (63.6%)	
Prior pacemaker or ICD care				
In-person device interrogation in past 1 year, *n* (%)	752 (68.5%)	732 (67.4%)	1471 (67.5%)	0.009
Time since device placement, median [IQR], years	3.33 [1.72, 5.57]	3.48 [1.74, 5.62]	3.35 [1.68, 5.53]	0.038
Time since last transmission, median [IQR], days	263 [158, 432]	234 [145, 412]	237 [152, 399]	0.032
Generator manufacturer, *n* (%)				0.055
Abbott	318 (29.0%)	291 (26.8%)	574 (26.4%)	
Biotronik	66 (6.0%)	71 (6.5%)	154 (7.1%)	
Boston Scientific	214 (19.5%)	224 (20.6%)	415 (19.1%)	
Medtronic	500 (45.5%)	500 (46.0%)	1035 (47.5%)	

*IQR* interquartile range, *ICD* implantable cardioverter-defibrillator

^a^Standardized mean differences between postcard vs. control arms in wave 1. A value < 0.1 denotes successful randomization

^b^Totals may not equal 100% because of the following numbers of unknown or missing variables: sex (*n* = 2), race (*n* = 299), ethnicity (*n* = 158), region (*n* = 5), rural (*n* = 4), and marital status (*n* = 13)

^c^States by region: Northeast (Connecticut, Maine, Massachusetts, New Hampshire, Rhode Island, Vermont, New Jersey, New York, Pennsylvania), Midwest (Illinois, Indiana, Michigan, Ohio, Wisconsin, Iowa, Kansas, Minnesota, Missouri, Nebraska, North Dakota, South Dakota), South (Delaware, District of Columbia, Florida, Georgia, Maryland, North Carolina, South Carolina, Virginia, West Virginia, Alabama, Kentucky, Mississippi, Tennessee, Arkansas, Louisiana, Oklahoma, Texas), West (Arizona, Colorado, Idaho, Montana, Nevada, New Mexico, Utah, Wyoming, Alaska, California, Hawaii, Oregon, Washington)

**Table 2:** Baseline Characteristics of Patient Population in Wave 2, by Randomization Arm

Characteristics	Mailed warning postcard (*n* = 1723)	Mailed encouraging postcard (*n* = 1750)	Standardized mean differences^a^
Demographic characteristics			
Age, median [IQR], years	70.9 [65.7, 76.9]	70.7 [64.8, 76.7]	0.050
Male sex, *n* (%)	1687 (97.9%)	1711 (97.8%)	0.002
Race, *n* (%) ^b^			0.014
American Indian or Alaska native	13 (0.8%)	13 (0.7%)	
Asian	8 (0.5%)	7 (0.4%)	
Black	289 (16.8%)	296 (16.9%)	
Native Hawaiian or other Pacific Islander	10 (0.6%)	9 (0.5%)	
White	1301 (75.5%)	1321 (75.5%)	
Ethnicity, *n* (%)^b^			0.026
Hispanic/Latinx	64 (3.7%)	57 (3.3%)	
Not Hispanic/Latinx	1596 (92.6%)	1638 (93.6%)	
Geographic region, *n* (%)^b,c^			0.028
Northeast	179 (10.4%)	192 (11.0%)	
Midwest	402 (23.3%)	394 (22.5%)	
South	825 (47.9%)	832 (47.5%)	
West	316 (18.3%)	331 (18.9%)	
Rural residence, *n* (%)^b^	654 (38.0%)	681 (38.9%)	0.020
Marital status, *n* (%)^b^			0.063
Married	839 (48.7%)	892 (51.0%)	
Divorced/separated	573 (33.3%)	576 (32.9%)	
Never married/single	142 (8.2%)	119 (6.8%)	
Widow/widower	163 (9.5%)	160 (9.1%)	
Co-morbidities, *n* (%)			
Coronary artery disease	1363 (79.1%)	1399 (79.9%)	0.021
Heart failure	1281 (74.3%)	1300 (74.3%)	0.001
Atrial fibrillation	1015 (58.9%)	1029 (58.8%)	0.002
Stroke	312 (18.1%)	329 (18.8%)	0.018
Dementia	211 (12.2%)	213 (12.2%)	0.002
Depression	670 (38.9%)	712 (40.7%)	0.037
Post-traumatic stress disorder	360 (20.9%)	383 (21.9%)	0.024
Alcohol use disorder	226 (13.1%)	210 (12.0%)	0.034
Cancer	352 (20.4%)	367 (21.0%)	0.013
Pacemaker or ICD characteristics			
Device type, *n* (%)			0.051
Pacemaker	684 (39.7%)	652 (37.3%)	
ICD	1039 (60.3%)	1098 (62.7%)	
Prior pacemaker or ICD care			
In-person device interrogation in past 1 year, *n* (%)	1032 (59.9%)	976 (55.8%)	0.084
Time since device placement, median [IQR], years	3.24 [1.63, 5.47]	3.43 [1.73, 5.64]	0.086
Time since last transmission, median [IQR], days	177 [140, 312]	184 [141, 346]	0.012
Generator manufacturer, *n* (%)			0.043
Abbott	452 (26.2%)	460 (26.3%)	
Biotronik	120 (7.0%)	107 (6.1%)	
Boston Scientific	365 (21.2%)	360 (20.6%)	
Medtronic	786 (45.6%)	823 (47.0%)	

*IQR* interquartile range, *ICD* implantable cardioverter-defibrillator

^a^Standardized mean differences between encouraging vs. warning postcard arms in wave 2. A value < 0.1 denotes successful randomization

^b^Totals may not equal 100% because of the following numbers of unknown or missing variables: sex (*n* = 2), race (*n* = 206), ethnicity (*n* = 118), region (*n* = 2), rural (*n* = 1), and marital status (*n* = 9)

^c^States by region: Northeast (Connecticut, Maine, Massachusetts, New Hampshire, Rhode Island, Vermont, New Jersey, New York, Pennsylvania), Midwest (Illinois, Indiana, Michigan, Ohio, Wisconsin, Iowa, Kansas, Minnesota, Missouri, Nebraska, North Dakota, South Dakota), South (Delaware, District of Columbia, Florida, Georgia, Maryland, North Carolina, South Carolina, Virginia, West Virginia, Alabama, Kentucky, Mississippi, Tennessee, Arkansas, Louisiana, Oklahoma, Texas), West (Arizona, Colorado, Idaho, Montana, Nevada, New Mexico, Utah, Wyoming, Alaska, California, Hawaii, Oregon, Washington)

### Primary Outcome

Of the 5657 patients who were mailed a postcard, 2756 (48.7%) sent an RM transmission within 70 days, compared to 530 (24.3%) of 2178 controls. The absolute difference between the intervention and control arms was 24.4% (95% CI 22.2%, 26.6%). When compared to controls, the odds of sending an RM transmission was 2.19 (95% CI 1.91, 2.50) times greater among patients who were mailed a “warning” postcard and 2.10 (95% CI 1.84, 2.41) times greater among patients who were mailed an “encouraging” postcard (Table 3). There was no significant difference in the primary outcome by messaging strategy (odds ratio [OR] for “warning” vs. “encouraging” message: 1.04, 95% CI 0.92, 1.18). Results were similar in the as-treated analysis (i.e., excluding patients whose postcards were returned as undeliverable) and in the sensitivity analysis excluding patients who died or were changed to non-active status.

**Table 3:** Association of Postcard Mailing with Transmission in 70 days

	Mailed warning postcard (*n* = 2821)	Mailed encouraging postcard (*n* = 2836)	Controls (*n* = 2178)	Odds ratio (95% CI) for warning postcard vs. controls	Odds ratio (95% CI) for encouraging postcard vs. controls	Odds ratio (95% CI) for warning vs. encouraging postcard
Primary outcome, remote monitoring transmission within 70 days of postcard mailing, *n* (%) patients^a^						
Intention-to-treat analysis^b^	1389 (49.2)	1367 (48.2)	530 (24.3)	2.19 (1.91, 2.50)	2.10 (1.84, 2.41)	1.04 (0.92, 1.18)
As-treated analysis^c^	1371 (50.9)	1349 (49.6)	530 (24.3)	2.44 (2.13, 2.81)	2.32 (2.02, 2.66)	1.05 (0.93, 1.20)
Excluding status changes^d^	1363 (50.4)	1346 (49.8)	520 (24.7)	2.21 (1.92, 2.53)	2.17 (1.89, 2.48)	1.02 (0.90, 1.16)

^a^Odds ratios reported for the primary outcome are derived from logistic regression using generalized estimating equations to account for repeated measures in the controls in wave 1 who were subsequently included in wave 2. All models control for wave number

^b^*N* = 7835 observations

^c^*N* = 7591 observations after excluding patients whose postcards were returned as non-deliverable

^d^*N* = 7516 observations after excluding patients who died or were changed to non-active remote monitoring status during the 70-day follow-up

Among the 2756 patients who received a postcard in waves 1 and 2 and sent an RM transmission within 70 days, 1979 (71.8%) patients transmitted after receiving the first postcard. A total of 777 (28.2%) patients transmitted only after receiving a second postcard; 28.9% in the “encouraging” group vs. 27.5% of patients in the “warning” group, absolute difference 1.4% (95% CI − 2.0%, 4.8%).

### Subgroup Analysis

There was a consistent benefit of both postcards on the primary outcome across most of the subgroups studied, with the exception of dementia and depression (Supplementary Figs. 1 and 2). Patients with dementia did not appear to benefit from either postcard, in contrast to patients without dementia (OR 95% CI for “warning” vs. controls: 1.10 (0.75, 1.62) vs. 2.42 (2.09, 2.80), *p*-value for interaction: < 0.001; OR 95% CI for “encouraging” vs. controls: 1.19 (0.81, 1.74) vs. 2.29 (1.98, 2.65), *p*-value for interaction: < 0.001). Patients with depression appeared to have a smaller benefit from the “warning” postcards, compared to those without depression, although they still derived benefit (OR 95% CI for “warning” vs. controls: 1.93 (1.56, 2.39) vs. 2.37 (1.99, 2.82), *p*-value for interaction: 0.049). Depression did not modify the effect of the “encouraging” postcards (*p*-value for interaction: 0.236).

## DISCUSSION

In this RCT, we found that mailing informational postcards doubled (24.4% absolute increase) RM transmissions among more than six thousand Veteran patients with pacemakers and ICDs who were previously non-adherent. Even as digital devices and artificial intelligence are revolutionizing the delivery of healthcare, results from this study demonstrate that simple solutions can still have a large impact. Because RM reduces hospitalizations, ICD shocks, and death, these findings suggest these postcards could make it more likely that patients achieve important clinical outcome benefits. We found no difference in the primary outcome between the two messaging strategies. Patients who received a “warning” message (loss-frame) and those who received an “encouraging” (gain frame) message were just as likely to send a transmission within 70 days. These results are consistent with prior literature that has not shown a consistent advantage of either strategy.^38–40^

In subgroup analyses, depression modified the effect of the “warning” postcard but not the “encouraging” postcard; patients with depression appeared to derive less benefit from the loss-frame messaging compared to patients without depression. Patients with dementia did not appear to benefit from either postcard. In prior research, we found that depression and dementia were associated with lower odds of adherence to RM.^27^ Otherwise, there was a consistent benefit of the postcard intervention across all remaining subgroups examined, including those with longer times since their last RM transmission. We also found that sending a second, reinforcing reminder postcard was important since more than one-fourth of RM transmissions occurred after the second postcard. These findings are consistent with several studies in other care contexts, such as vaccination, which have shown benefits of sending multiple reminders.^42–44^

Most interventions to improve adherence to RM among patients with pacemakers and ICDs have focused on mobile apps studied in non-randomized observational studies. In one study, patients who installed a mobile app providing information on communicator setup, troubleshooting, and connection status had higher rates of being in a monitored status at 3 months.^45^ An observational study using matched historical controls found that a mobile app paired with Bluetooth-enabled devices improved RM transmissions.^46^ Another study demonstrated an increase in the proportion of patients who sent a remote transmission in the year after pacemaker/ICD implantation by providing a smartphone app, although the authors noted the lack of a control group and that part of the observed effect might have been due to increased adoption of RM over time.^47^ While these mobile-based strategies can support RM adherence, our RCT demonstrates the benefits of a direct mailing strategy to patients. This is particularly relevant for Veteran patients, given at least 10% of those engaged with the VA’s patient portal do not regularly use smartphones.^48^ For Veterans with smartphones, there is potential to integrate app-based interventions with direct mailings. Given that approximately half of patients still did not send transmissions despite the two postcard mailings, it is clear that there is a need for multi-level interventions to support adherence.

Recent cardiology professional society consensus recommendations place even greater importance on RM, suggesting that patients who are adherent to RM need to only follow-up in-person every 2 years (which is longer than current recommendations).^2^ Further, these recommendations suggest that patients may not need to have any routine in-person or remote transmissions as long as the patient’s pacemaker or ICD is consistently and continuously connected to RM.^2^ For these possibilities to be successful, patients must be adherent to RM; if they become non-adherent, they need to be quickly identified and receive an intervention to restore adherence. Our study showed that even when patients have been non-adherent for longer periods of time, the postcards were still effective in restoring adherence.

Our study has several strengths. This was a large national study conducted among all eligible Veterans followed by the VANCDSP across the USA with pacemakers and ICDs from all major manufacturers. We also used an implementation science approach focused on closing the performance gap between professional society consensus and practice through the most rigorous design, an RCT, which demonstrated the causal impact of the postcard intervention. This study thus addresses an important gap as RCTs evaluating health care delivery strategies have historically been rare.^49^ Another strength of the study is the relative simplicity of the intervention and that it does not require clinician time, which is already strained by the workload of managing device clinics^29^ and an often high number of alerts.^50^ The postcards go directly to patients and leverage support from the device manufacturers by providing the customer service number, as these companies have dedicated help lines that assist patients in troubleshooting RM challenges and can mail additional equipment needed for RM, if necessary.

A final notable impact is that this study led to operational changes; the VANCDSP now sends an average of 1800 monthly postcards, including up to one reminder for patients who have missed an RM transmission. Additionally, if patients become adherent but then again become non-adherent, the VANCDSP will again send them up to two postcards.

The study should also be considered in the context of its limitations. First, we did not include Veterans in developing or reviewing the postcards; postcard content was developed by the author team. Veteran engagement could have strengthened the work, and we have now engaged Veterans in development of subsequent similar interventions. Although we excluded patients who were randomized and transmitted prior to postcard mailing, a small number of patients could have transmitted during the few days between the mailing and actual receipt of postcards by intervention patients. However, this should not have affected results of the trial unless there was a difference in the proportion of intervention vs. control patients who transmitted after postcards were mailed; any difference would be unexpected given the randomization and intent-to-treat approach. The study was conducted during the COVID-19 pandemic, which led to reductions in in-person evaluations, often in favor of RM. However, those changes occurred in March/April 2020, and postcard mailing began in August 2020, so all intervention and control patients had experienced at least one transmission cycle prior to the start of the study. It is possible that patients were more responsive to the postcards because of the emphasis on remote care during the pandemic, but the response rate in ongoing clinical practice is similar to or better than that seen in this RCT. Another limitation is that the study population was largely male and English-speaking, and thus our results may not generalize to other populations. Finally, we excluded patients who had never sent RM transmissions, who we assumed to have higher barriers to RM and would have necessitated different text within the postcard, as well as patients who did not have wireless-capable devices.

Future studies should examine additional strategies to engage patients who did not respond to the postcard intervention and to understand why they did not. Future studies should also examine whether digital reminders, which are faster, potentially less expensive, and able to correct non-adherence more quickly, achieve similar improvements in adherence for some patients and determine the optimal combination of digital and postcard reminders.

## CONCLUSION

Informational postcards significantly improved adherence to RM among Veteran patients with pacemakers and ICDs who were previously non-adherent to RM. This simple intervention could improve access to RM care and has potential to improve clinical outcomes.

### Supplementary Information

Below is the link to the electronic supplementary material.

Supplementary file1 (DOCX 513 KB)
